# FERONIA: A Receptor Kinase at the Core of a Global Signaling Network

**DOI:** 10.1146/annurev-arplant-102820-103424

**Published:** 2024-07-02

**Authors:** Alice Y. Cheung

**Affiliations:** Department of Biochemistry and Molecular Biology, Molecular Biology Program, Plant Biology Graduate Program, University of Massachusetts, Amherst, Massachusetts, USA;

**Keywords:** growth, reproduction, survival, glycosylphosphatidylinositol-anchored protein, GPI-AP, RAPID ALKALINIZATION FACTOR, RALF, RAC/ROP, ROS, extracellular matrix, cytoplasmic pathway, nuclear pathway

## Abstract

Initially identified as a key regulator of female fertility in *Arabidopsis*, the FERONIA (FER) receptor kinase is now recognized as crucial for almost all aspects of plant growth and survival. FER partners with a glycosylphosphatidylinositol-anchored protein of the LLG family to act as coreceptors on the cell surface. The FER-LLG coreceptor interacts with different RAPID ALKALINIZATION FACTOR (RALF) peptide ligands to function in various growth and developmental processes and to respond to challenges from the environment. The RALF-FER-LLG signaling modules interact with molecules in the cell wall, cell membrane, cytoplasm, and nucleus and mediate an interwoven signaling network. Multiple FER-LLG modules, each anchored by FER or a FER-related receptor kinase, have been studied, illustrating the functional diversity and the mechanistic complexity of the FER family signaling modules. The challenges going forward are to distill from this complexity the unifying schemes where possible and attain precision and refinement in the knowledge of critical details upon which future investigations can be built. By focusing on the extensively characterized FER, this review provides foundational information to guide the next phase of research on FER in model as well as crop species and potential applications for improving plant growth and resilience.

## 1. INTRODUCTION

**Figure 1:**
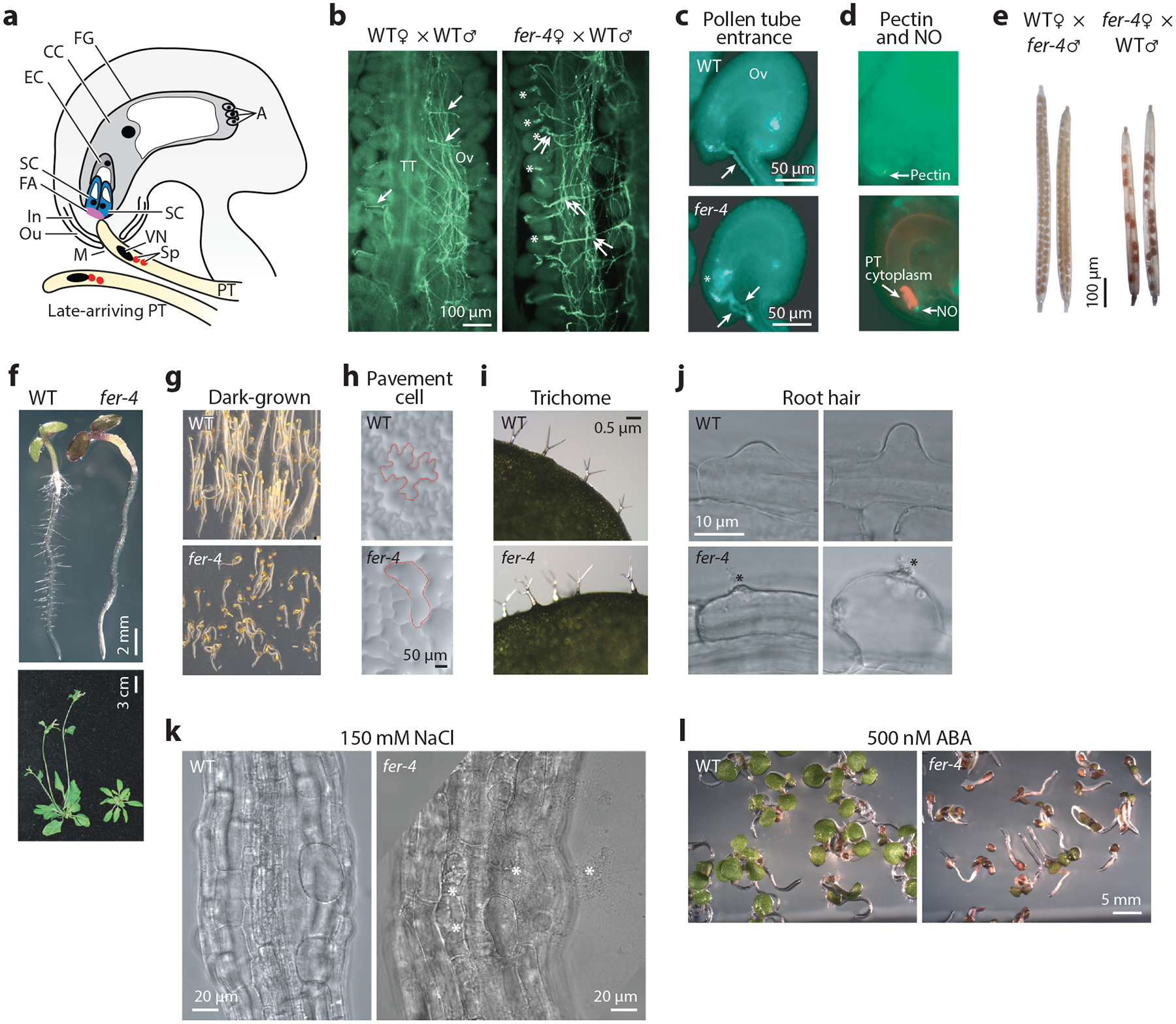
The multitasking FER. Loss of FER in *Arabidopsis* induces a plethora of growth and reproduction phenotypes. An abbreviated collection of these phenotypes is shown here. (*a*–*e*) Reproduction phenotypes (22, 24, 59, 108). (*a*) A diagram of PT–Ov interaction. PTs target Ovs to penetrate the FG, each transporting two Sp cells in its cytoplasm. The CC is the precursor to the seed endosperm. SCs produce attractants, and the first-arriving PT enters one of the SCs; late-approaching PTs are deterred and instead enter other not-yet-penetrated Ovs. The FA is a thickened cell wall region secreted by the synergids. In and Ou are precursors of the seed coat. M is the ovular aperture targeted by the pollen tube. (*b*) PT growth in WT or *fer* pistils. In WT pistils, a single PT exits from the main growth path in the TT to target Ovs one at a time, and each bursts to release sperm upon penetrating the FG. In *fer* pistils, the one PT:one Ov pattern is perturbed, and bundles of 2 to 3 PTs exit from the TT. Multiple PTs penetrate a single *fer* Ov but fail to burst, resulting in the PT pileup phenotype. (*c*) Individual Ovs showing a single burst PT in a WT Ov and a pileup of unburst PTs in *fer* Ovs. (*d*, *top*) A WT Ov stained for de-esterified pectin and (*bottom*) a WT Ov stained for NO and the SCs are filled with the cytoplasm from a just-penetrated and -burst Tomato-labeled PT. (*e*) Siliques from reciprocal crosses show a (*left*) WT pistil pollinated by *fer* pollen with a full seed set, but (*right*) seed yield is reduced in a *fer* pistil pollinated by WT pollen. (*f*–*l)* Growth defects and stress sensitivity of *fer* seedlings (23, 31, 74). (*f)* Growth phenotypes. (*Top*) The most severe seedling defect is growth arrest; (*bottom*) when not arrested, growth to maturity is consistently delayed. (*g*, *top*) Etiolated WT seedlings grown in the dark; (*bottom*) *fer* seedlings de-etiolate in the dark and are compromised in gravitropism. (*h*) *fer* leaf epidermal pavement cells lack the jigsaw puzzle shape characteristic of WT pavement cells. Auxin and RAC/ROP signaling are both required for the pavement cell shape differentiation (29, 98, 99). (*i*) *fer* trichomes are deformed. (*j*) *fer* root hairs burst, and cytoplasm is often seen leaking out of the root hair cells. Mutant roots lack ROS, a result of suppressed RAC/ROP-controlled, NADPH oxidase–dependent ROS production. The RAC/ROP-to-ROS pathway is crucial for polarized cell growth (29, 33, 98, 99). *fer* roots are not responsive to auxin-stimulated ROS increase and auxin-stimulated root hair development (23). (*k*) Under high-salt conditions, root cells of *fer* seedlings burst (A. Cheung, unpublished observations) and root growth is arrested (31). (*l*) Germinating *fer* seedlings are hypersensitive to ABA and fail to turn green. *llg1* mutants phenocopy *fer* seedlings; *lre* mutants phenocopy *fer* reproduction phenotype (74). Abbreviations: A, antipodal cells; ABA, abscisic acid; CC, central cell; EC, egg cell; FA, filiform apparatus; FER, FERONIA; FG, female gametophyte; In, inner integument; M, micropyle; Ou, outer integument; Ov, ovule; PT, pollen tube; ROS, reactive oxygen species; SC, synergid cell; Sp, sperm; TT, transmitting track; VN, vegetative cell nucleus of the pollen tube; WT, wild-type. Panels *a* and *e* adapted from Reference 22, panels *b*–*d* adapted from Reference 24, and panels *f*–*j* and *l* adapted from Reference 74.

In 2003, two different mutations in the same *Arabidopsis* gene were reported, each inducing female-sterile mutants with a dramatic ovule phenotype with a huge pileup of pollen tubes inside the female gametophyte where fertilization takes place (Figure 1). One study named the responsible gene *SIRÉNE* after the mermaid fairy (108) and the other *FERONIA* (*FER*) after the Etruscan goddess of fertility (59). *FER* was used almost exclusively in later studies and was determined in 2007 to encode a receptor kinase with the typical configuration of an extracellular domain (ECD), a single transmembrane span, and a cytoplasmic kinase domain with self-phosphorylation activity (26). Subsequent studies have shown that FER plays many important roles in plant growth and survival (13, 23, 49, 129, 152), providing a rich ground for plant signaling research.

**Figure 2:**
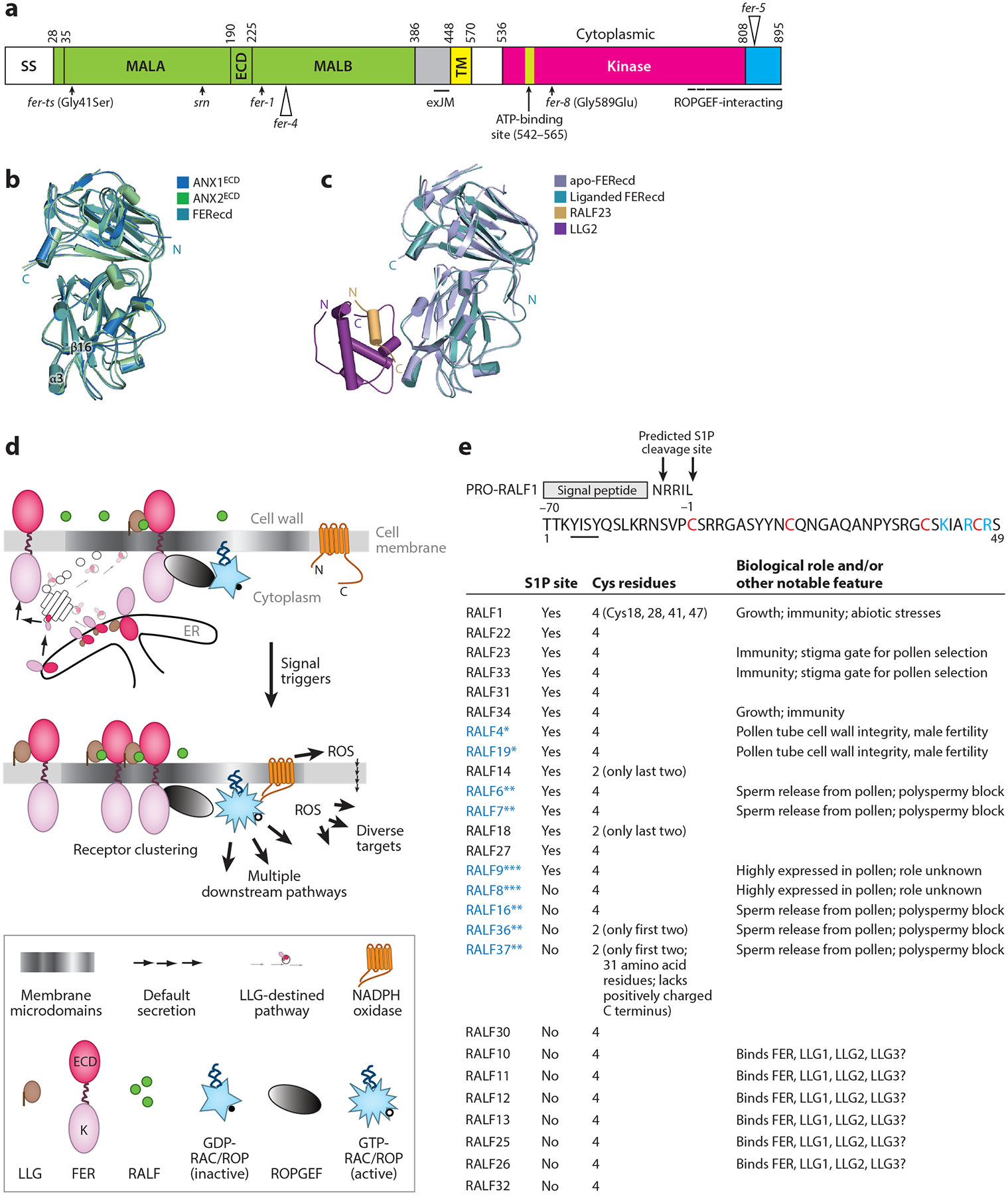
Core components of the FER signaling module. (*a*) Domain map of FER, representative of the FER family receptor kinases. Tandem Malectin-like domains are designated MALA and MALB. Alleles referred to in the text are indicated. Gly41Ser in *fer-ts* (67) is analogous to Gly37Asp and Glu150Lys conversion in MALA and MALB, respectively, in *the1-1* and *the1-2* mutations in THESEUS1 (54). The ROPGEF-interacting domain spans the C-terminal half of the FER cytoplasmic domain (23). *fer-8* is defective in the FER-to-ROS pathway (126). LLG1 binds the exJM (74). (*b*) Three-dimensional structures of the ANX1^ECD^, ANX2^ECD^, and FERecd show similar architecture. (*c*) The ECD of apo-FER and of the RALF23-LLG2-FERecd heterocomplex. (*d*) A model of the FER-RAC/ROP-ROS signaling pathway. LLG is postulated to chaperone and deliver LLG-bound FER from the ER to the functional location for the FER-LLG coreceptor pair, presumably in the LLG-destined membrane microdomain. Unbound FER is transported through the default secretory pathway to the cell membrane, where it remains inactive. Signals trigger ligand-activated FER to the RAC/ROP GTPase pathway, which impacts diverse cytoplasmic response pathways. (*e*) Selected *Arabidopsis* RALF peptides and their amino acid sequence and functional features. A subset of RALFs are expressed as prepro-peptides with a predictive cleavage site for S1P (1, 145). The top panel shows not-yet-processed RALF1, a prototypical RALF peptide (1). Most RALF peptides have four Cys residues; others have two as indicated (1). Abbreviations: ANX, ANXUR; ATP, adenosine triphosphate; ECD, extracellular domain; ER, endoplasmic reticulum; exJM, extracellular juxtamembrane region; FER, FERONIA; FERecd, FERONIA extracellular domain; GDP, guanosine diphosphate; GTP, guanosine triphosphate; K, kinase; LLG, LORELEI-like glycosylphosphatidylinositol-anchored protein; MALA, Malectin-like A; MALB, Malectin-like B; RALF, RAPID ALKALINIZATION FACTOR; ROPGEF, ROP-guanine nucleotide exchange factor; ROS, reactive oxygen species; S1P, SITE-1 PROTEASE; *srn*, *sirène*; SS, signal peptide; TM, transmembrane. Panels *a* and *d* adapted from Reference 74, panels *b* and *c* adapted from Reference 145, and panel *e* includes data from References 1 and 145.

FER belongs to a 17-member receptor kinase family in *Arabidopsis* and is conserved across plants (6, 11, 53, 147). Most *Arabidopsis* FER family proteins, many also named after deities, have been functionally and mechanistically examined to various extents (Table 1). As a family, they are often referred to as *Catharanthus roseus* receptor-like kinase 1–like (CrRLK1-like) (116).^1^ Recent literature has adopted Malectin-like or Malectin/Malectin-like domain–containing receptor-like kinases (RLKs) as designations (101, 147) based on the homology of their ECDs with the animal diglucose-binding protein Malectin (38, 110, 111) (Figure 2), which distinguishes them from other members of the plant super RLK family (123). FER and FER-related are used for general referrals here.

**Figure 3:**
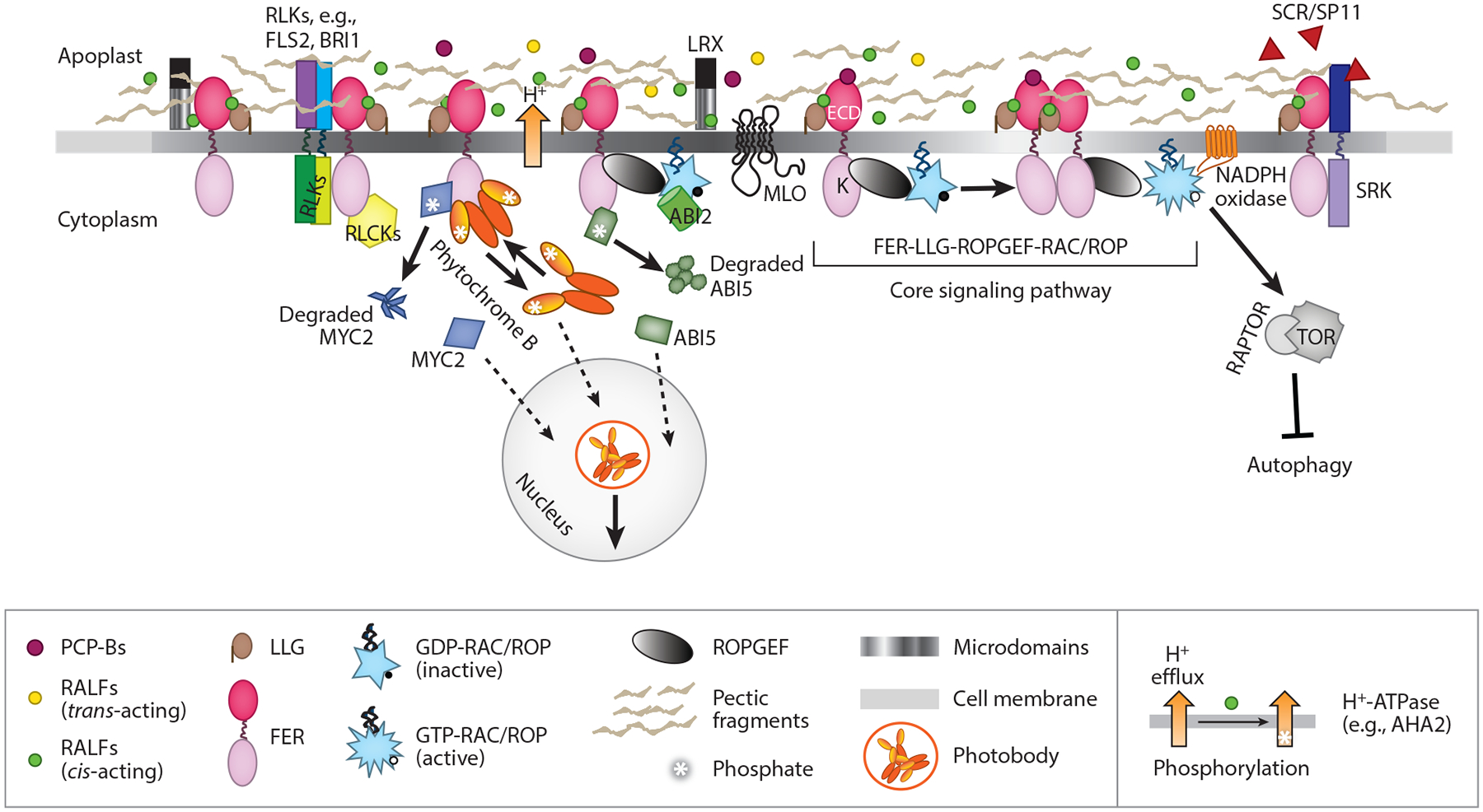
The core FER signaling pathway and its elaborators along the cell surface. The FER-LLG1–ROPGEF–RAC/ROP signaling pathway is considered the core of the broader FER-LLG1 signaling network. Elaborators from the extracellular matrix, or the apoplast, to the cytoplasm and nucleus discussed in the text (see Sections 4 and 5) are depicted. RLKs (*left*) collectively refer to FLS2, BRI1 (129), and potentially additional receptor kinases, such as stigma-located SRK (*right*), whose interaction with FER, also a receptor kinase, is regulated in *trans* by pollen *S*-factor SCR/SP11 and impacts the FER-to-ROS pathway (57). NADPH oxidase, which produces ROS, is one of several RAC/ROP effectors and has been demonstrated to mediate several FER-controlled processes. ABI2 integrates into the FER core pathway via its interaction with RAC/ROPs (152). H^+^-ATPase is central to RALF-regulated growth (4, 103). The schematics for H^+^-ATPase are intended to demonstrate that (*i*) RALF-induced inhibition of H^+^-efflux induces medium alkalinization, and this aligns with the growth inhibitory activity of RALFs (4, 103), and (*ii*) RALF1 triggers the phosphorylation of AHA2 (52). Molecular and biochemical interactions providing causal linkages for the RALF-signaled H^+^-ATPase phosphorylation remain to be established. Dashed arrows indicate translocation. Abbreviations: ABI, Abscisic acid–insensitive; AHA2, *Arabidopsis* H^+^-ATPase 2; BRI1, Brassinosteroid Insensitive 1; ECD, extracellular domain; FER, FERONIA; FLS2, Flagellin-Sensing 2; K, kinase; LLG, LORELEI-like glycosylphosphatidylinositol-anchored protein; LRX, leucine-rich repeat extensin-like; MLO, Powdery Mildew Resistance Locus O; MYC, a bHLH (basic helix-loop-helix) transcription factor; PCP, Pollen Coat Protein; RALF, RAPID ALKALINIZATION FACTOR; RLCK, receptor-like cytoplasmic kinase; RLK, receptor-like kinase; ROPGEF, ROP-guanine nucleotide exchange factor; ROS, reactive oxygen species; SCR/SP11, S-Locus Cysteine-Rich/S-Locus Protein 11; SRK, S-Locus Receptor Kinase; TOR, TARGET OF RAPAMYCIN.

Many reviews, perspectives and commentaries (e.g., 10, 15, 32, 42, 73, 75, 84, 101, 135, 147, 159) provide a comprehensive overview and a running log of the rapidly unfolding field. Here, I provide historical and bird’s-eye views of foundational aspects of FER signaling and mechanisms uncovered thus far as well as findings from several other FER-related receptors. The goal is to provide a thread connecting the core components of FER signaling with factors from the extracellular matrix to the nucleus that elaborate its underlying molecular mechanisms and diversify FER signaling (Figure 3). The various functional roles played by FER are discussed to provide the biological context for how individual components contribute to FER signaling. I discuss this foundational information to help chart the future course of investigations to advance mechanistic understanding and inform the development of strategies for improving the growth and resilience controlled by the broader FER family receptor kinases in crop species (e.g., 57, 107, 149, 153, 157).

## 2. THE DISCOVERY OF FERONIA: FROM THE GODDESS OF FERTILIY TO THE QUEEN OF PROMISCUITY

The discovery of FER as a receptor kinase (26) coincided with the report of the animal protein Malectin, named for its capacity to bind maltose (110, 111), and the identification of the FER-related THESEUS1 as having a role in sensing cell wall damages (54). ECD homology with Malectin led to intense speculation that the FER family receptor kinases could be sensors of perturbations in the cell wall (6, 11, 53). THESEUS1 was pivotal in promoting this hypothesis since it was identified as a suppressor of growth defects in cell wall mutants deficient in cellulose. Several studies that focused on growth, development, or immunity responses to pathogens coalesced onto FER, establishing it as a broadly functioning regulator of growth and survival under normal conditions and in challenging times (13, 23, 49, 120, 129, 152).

### 2.1. The Discovery of FER and Its Function in Female Fertility

The founding heterozygous FER mutants, *fer-1*/+ and *sirène*/+ (59, 108), and the later identified homozygous *fer-4* mutant (22, 24) display severe female fertility defects (Figure 1).^2^ In flowering plants, two immotile sperm cells are transported as cytoplasmic cargoes by pollen tubes growing inside the pistil, each targeting an ovule and penetrating the female gametophyte within (10, 62, 64) (Figure 1a–c). Once inside the female gametophyte, the pollen tube bursts to release the sperm cells for fertilization. Plants have evolved mechanisms to suppress polyspermy, the fertilization of an egg by multiple sperm cells (127); thus, rarely are wild-type ovules penetrated by more than one pollen tube (Figure 1b,c). Typically, in 80% of *fer* mutant female gametophytes, the penetrated pollen tube continues to grow and fails to burst and release sperm, thus precluding fertilization and seed production (Figure 1d). Additionally, half of the *fer* ovules are also penetrated by multiple pollen tubes, and their continued growth gives rise to a dramatic pollen tube pileup inside the female gametophyte (Figure 1b,c).^3^ Prevention of supernumerary pollen tube entrance is controlled by FER on two levels: one governing pollen tube exits from its main growth axis to target the ovules (Figure 1b) and the other locally at the ovule (Figure 1c) (24).

### 2.2. From the Goddess of Fertility to the Multitasking FER with a Global Role in Growth and Survival

Several studies focusing on different biological processes have converged on FER, revealing its remarkably broad functional involvements in growth and development (Figure 1). These include brassinosteroid (BR)- and ethylene-regulated growth and development and the functional intersection between FER and BR-regulated growth (13, 49). An effort to identify cell surface regulators of polarized cell growth identified FER as a major regulator of root hair growth and the loss-of-function *fer-4* mutant as pleiotropic (23, 74) (Figure 1). These findings implicated FER intersections of several auxin-regulated processes such as reactive oxygen species (ROS) production in root and root hairs (23), gravitropic response (16), and epidermal pavement cell shape morphogenesis (74, 76). FER has also been implicated in mechanical sensing (25, 83, 120), impacting cellular metabolism (e.g., 86, 146, 148), contributing to flowering time and seed size (138, 151), and mediating the balancing acts between TOR signaling and autophagy (102, 125, 140).

FER is important for stress management (Figure 1). Its functions intersect with abscisic acid (ABA), often regarded as a stress hormone. *fer* seedlings are hypersensitive to ABA-suppressed seedling growth (74, 139, 152) and defective in stomatal aperture closure regulation (154). FER buffers against high salinity and protects seedlings from photooxidative damages (31, 80, 121) and is also a critical regulator of immunity responses (50, 101, 129, 159). Interestingly, an effort to identify plant genes that regulate the rhizosphere microbiome discovered that FER participates in regulating soil microbial species; the microbes in turn impact plant health (126).

## 3. CORE COMPONENTS OF FERONIA SIGNALING

A FER signaling module on the cell surface consists of three core components (Figure 2; Table 1). FER partners with a glycosylphosphatidylinositol-anchored protein (GPI-AP) from the LORELEI-like GPI-AP (LLG) family (100); together they function as a coreceptor pair for signal perception (22, 73, 74). Several peptide growth regulators from the RAPID ALKALINIZATION FACTOR (RALF) family (1, 4) bind to FER-LLG1 and regulate its signaling from the cell surface (10, 32). For signal mediation to the cytoplasm, FER interacts directly with ROPGEFs, the guanine nucleotide exchange factors that activate RAC/ROPs, the RHO GTPases and major molecular switches of plants that mediate myriad processes (29, 33, 98, 99). FER is almost ubiquitously expressed at a relatively high level throughout the plant life cycle, except in pollen (23, 26).^4^ The LLG family is comprised of 4 members: LORELEI, the founding member, is expressed almost exclusively in the female gametophyte (9, 81, 133); LLG1 is broadly expressed in vegetative tissues and in sporophytic tissues of the pistil (74, 77, 133); and LLG2 and LLG3 are pollen specific (41, 100). The RALF family has more than 35 members (1). The expression of RALFs is often redundant, and most do not show notable cell- or tissue-specificity, except for several that are highly expressed in pollen (1, 41). There are 14 ROPGEFs and 11 RAC/ROPs in *Arabidopsis* (33, 99). Combinatorial use of coexpressed core components harnesses tremendous signaling potential. The discussion here focuses on the FER-LLG1 coreceptor pair and its best-characterized signaling ligands RALF1 and RALF23, highlighting mechanistic insights.

### 3.1. FER, a Malectin-Like Receptor Kinase

FER is located in the cell membrane. Its extracellular Malectin-like domains and diverse intracellular signaling pathways have been extensively reviewed (6, 11, 53, 73, 75, 101, 147, 159). Malectin is a diglucose-binding protein located in the lumen of the mammalian endoplasmic reticulum (ER) (38, 110, 111). The ECDs of the FER family receptor kinases show tandem domains [Malectin-like A (MALA) and MALB] (Figure 2a–c) with low levels of sequence homology with Malectin (101, 147). This homology led to speculations of a carbohydrate-binding capacity for the FER family receptors and thus the potential to function as cell wall sensors, detecting perturbations in the cell wall and mediating the signals to elicit cytoplasm responses (6, 11, 53). Thus far, physical and/or functional interactions with the cell wall have been demonstrated for FER, the FER-related THESEUS1, and pollen-expressed ANXURs and Buddha’s Paper Seals (BUPSs) (7, 24, 30, 31, 54). The MAL domains in these receptor kinases are critical since mutations in either domain can independently compromise their functions (e.g., 54, 67, 145) (Figure 2a).

ECD-swapping experiments between FER and the related ANXUR1 and HERCULES1 showed that they are not interchangeable (65). While having the capacity to form heteromers with related receptor kinases (e.g., 77), FER is functionally self-sufficient for its biological roles; that is, phenotypes in loss-of-FER mutants are profound and unambiguous (13, 22–24, 59, 108, 129). FER-related receptor kinases also have a similar functional capacity as FER. For instance, ANJEA and HERCULES1 act redundantly in ovules. Similarly, double-mutant seedlings of *hercules1 theseus1*-*4*, a gain-of-function hypermorphic allele (89), mimic *fer* seedlings, albeit not as severely (49). Loss-of-FER-related CURVY, whose name refers to its distorted trichome phenotype, also induces loss of the jigsaw puzzle epidermal pavement cell pattern (34) documented in *fer* mutants (23, 74) (Figure 1h,i). On one hand, FER, with its broad expression profile and high expression levels, can be hypothesized to predominate and mask contributions from related receptors. On the other hand, loss-of-function *fer* mutations, such as the most prevalently used *fer-4*, are not fully penetrant (22–24, 74) in their reproductive defects,^5^ and mutant seedlings show a range of phenotype severity. This is possibly due to slight but mitigating fluctuations in cellular and environmental conditions during which activities from other FER-related receptors could provide adequate compensation.

Although FER acting alone is sufficient for its various biological roles, several FER-related receptor kinases show functional redundancy, and their activity depends on heteromer formation with related receptors. These properties are most evident in two pairs of pollen-predominant FER-related receptor kinases: ANXUR1 and ANXUR2 (7, 8, 91) and BUPS1 and BUPS2 (41, 43) (Table 1). Phylogenetically, ANXURs are most closely related to FER; BUPSs are in a different subclade that also includes the slightly diverged ERULUS, which is also expressed in root hairs and participates in polarized root hair and pollen tube growth (72, 114, 115). ANXUR1 and ANXUR2 function redundantly; single mutants appear normal while double *anxur1 anxur2* mutants are male sterile due to precocious disintegration of the mutant pollen tubes (8, 91). By contrast, there is a clear functional imbalance between BUPS1 and BUPS2; loss of BUPS1 resulted in >99% male sterility (41, 163), while loss of BUPS2 resulted in a phenotype that appeared perfectly normal. In vitro, the ECDs of ANXURs and BUPSs self-interact and form ANXUR/BUPS heteromers. This and the fact that loss of either the ANXURs or the BUPSs is adequate to induce male sterility in vivo are consistent with ANXURs and BUPSs functioning as obligatory heteromers (41, 42).

Studies of FER and its pollen-expressed counterparts suggest considerable functional complexity for these receptor kinases. One FER function in female fertility is inducing pollen tube disintegration upon arrival at the target female gametophyte to release sperm for fertilization (22). This is opposite to the function of the ANXURs and BUPSs in male fertility, which ensures that pollen tubes do not burst precociously during growth in the pistil (41). It is also different from the function of FER in supporting root hair growth and integrity (23). Moreover, a point mutation rendering an Ala385Val conversion in the MALB domain in ANXUR1 enhanced immunity response in four-week-old *Arabidopsis* plants but did not affect its pollen function (85). Interestingly, while Ala385 is conserved in ANXUR1 and ANXUR2, the corresponding analogous amino acid residue in FER is Val, but the wild-type ANXUR1 ECD could not functionally substitute that of FER (65). Whether the Ala385Val substitution in the ANXURs could contribute to an evolutionarily determining change resulting in divergent male and female functions between FER and ANXURs will be interesting to explore.

### 3.2. The LLG Family of GPI-APs as Coreceptors and Chaperones of FER: A Partnership Providing Functional Diversity Between Cell and Tissue Types

Two independent studies identified that mutations in *Lorelei* induced a pollen tube pileup and supernumerary pollen tube penetration phenotypes similar to those observed in *fer* ovules (9, 133). These findings laid the groundwork for the discovery of LLG family GPI-APs as coreceptors of FER (30, 43, 74).

#### 3.2.1. FER-LLG as coreceptors on the cell surface.

Focusing on LLG1, the only vegetative tissue-expressed member of the LORELEI/LLG1 family, Li et al. (74) showed that loss of LLG1 induces pleiotropic growth and developmental defects indistinguishable from those of *fer* mutant seedlings but that *llg1* mutants are normal in reproduction. Molecular interaction studies established that FER and LLG1 interact directly and on the cell surface and that LLG1 associates with FER and RALF1 in a tripartite complex. LLG1 and LORELEI are also in the same functional complex with FER and the RAC/ROP signaling module (22, 74, 77) (see Section 3.5). This evidence and demonstrations that RALF peptides bind both FER and LLG1 (52, 145) provide strong support for FER-LLG1 functioning as coreceptors that mediate diverse downstream signaling responses in vegetative tissue while FER-LORELEI functions in the female gametophyte. Partnering with differentially expressed LLG family proteins to form coreceptor pairs would seem an expedient strategy for FER family receptors to diversify signaling capacity throughout development.

#### 3.2.2. LLG1 chaperones FER, delivering FER-LLG1 to its functional location.

Cell membrane–associated GPI-APs are anchored on the outer leaflet of the lipid bilayer and located in steroid- and sphingolipid-enriched membrane microdomains (68, 150, 164). Also referred to as lipid rafts, these membrane subdomains are important for orchestrating or initiating signaling activities and serve as platforms where signaling molecules, such as receptors, concentrate. Li et al. (74) showed that FER and LLG1 interact in the ER, where they first encounter each other in the secretory pathway. FER–GREEN FLUORESCENT PROTEIN (GFP) expressed in *llg1* showed considerable retention of the receptor kinase in the ER, consistent with the idea that FER depends on LLG1 to exit the compartment. Interaction between LLG1 and a small juxtamembrane region on the extracellular side of the FER transmembrane span (exJM) (Figure 2a) is crucial for anterograde FER traffic from the ER.

Li et al. (74) postulated through their model that LLG1-bound FER piggybacks on the ER-exiting LLG1 to be delivered together to the GPI-AP-destined location where FER-LLG1 signaling is activated. It bears mentioning that while the ER retention of FER-GFP in *llg1* cells appeared overwhelming, it was nevertheless incomplete, and the presence of FER-GFP remained notable on the cell membrane (74). However, its presence in the cell membrane was not enough to activate FER activity, since *llg1* mutants fully phenocopy knockout *fer* mutants. Thus the original piggybacking model is updated (Figure 2d) to include that while the LLG1-bound FER is chaperoned to LLG1-destined membrane microdomains for activation of its signaling activity, the pool of non-LLG1-bound FER was presumably transported to the cell membrane via the default secretory pathway but remains inactive in the cell membrane.

#### 3.2.3. Other FER-LLG1-related partnerships.

Similar coreceptor partnerships exist for FER- and LLG1/LORELEI-related proteins (Table 1). Pollen-specific LLG2 and LLG3 bind the pollen-specific ANXURs and BUPSs. *llg2 llg3* double-mutant pollen tubes burst precociously, never arriving at the ovules, phenocopying the receptor kinase mutants (41, 43). LLG2 also binds to the exJM of ANXURs (30). The ECD of FER-related LETUM2, which mediates autoimmunity, also interacts with LLG1 and depends on it for transport to the cell membrane (58). LETUM2 was named in a series after LETUM1, another FER family receptor kinase that controls cell death during an immunity response (79, 101).

GPI-APs have long been considered important for orchestrating signaling from the cell surface (68, 150, 164), but precisely how they impact signaling is not well understood. Without a cytoplasmic domain, GPI-APs lack the capacity for signal mediation to the cytoplasm. Partnering with receptor kinases and enabling their delivery to membrane microdomains for activity activation could be among the underlying mechanisms that allow GPI-APs to function as enablers of signaling from the cell surface.

### 3.3. RALFs as Signaling Ligands for FER, Bringing to Light the Biological Roles of a Family of Peptide Growth Regulators

RALFs were first identified in the Solanaceae in 2001 and named for their ability to rapidly (in the first minutes of treatment) alkalinize plant culture media (104, 105). RALFs are conserved, small (~5 kDa), secreted growth regulatory peptides (1, 4). A mass spectrometry study in 2014 aimed at determining the impact of *Arabidopsis* RALF1 on its phosphoproteome (52) identified FER as its most prominently phosphorylated target. It also showed that RALF1 binds the FERONIA extracellular domain (FERecd) and that RALF1 biological activities, which include inducing cytoplasmic Ca^2+^ spikes and rapid medium alkalinization and inhibiting root growth, were dependent on FER. Together these established a signal–receptor relationship between RALF1 and FER. RALF1 also stimulated phosphorylation of the *Arabidopsis* H^+^-ATPase 2 (AHA2) (52) (Figure 3), which mediates H^+^ efflux, acidifying the apoplastic space and providing a major driving force for cell growth (103). These observations are consistent with a functional linkage between RALF1 and the proton pump, but the precise signaling relationship between the two remains unclear.

Most RALFs are expressed as precursor proteins; their pre- and prosequences are processed by signal peptidases and cleaved by the SITE-1 PROTEASE (S1P), respectively, before secretion of the mature peptides to the apoplast (1, 4, 128) (Figure 2e). A forward genetics study aimed at expanding the understanding of immunity signaling identified S1P as an important regulator of immunity responses, implicating RALFs in immunity signaling (129). One of the substrates of S1P is RALF23 (128), which binds FERecd and functions as a signaling ligand to suppress FER-dependent defense responses in host plants (discussed in Section 4.3.2).

RALF ligands for FER-related receptor kinases have also been reported (Table 1). RALF34 is a ligand for THESEUS1 (46). Male-female interactions that enable reproduction provide a rich ground for uncovering similar ligand-receptor relationships. In the pistil, stigma-expressed RALF33 functions with FER and a coexpressed FER-related receptor ANJEA. The RALF33-FER/ANJEA-LLG1 module acts as a gatekeeper on the stigma, differentiating between desirable and undesirable mates at the pollen-receptive surface (57, 77, 157). In the substigmatic tissues that support pollen tube growth, pollen-expressed RALF4 and RALF19 are ligands of ANXUR/BUPS-LLG2/LLG3 (41). Together the pollen-assembled modules maintain pollen tube integrity until it reaches an ovule and enters the target female gametophyte (30, 41, 43). RALF-receptor relationships are not strictly governed; that is, an individual RALF might target multiple receptors, and a receptor might respond to multiple RALFs (Figure 2e). It has been postulated that maturation through the S1P processing pathway could be a distinguishing feature in the capacity of some RALF peptides, for example, RALF23, RALF33, and RALF34, to regulate immunity response, while others, for example, RALF32, lack this capacity (129, 145).

### 3.4. The RALF-FER-LLG Tripartite Module: a Structural Perspective

Three crystallographic studies have independently generated high-resolution three-dimensional structures for ANXUR1, ANXUR2, and FERONIA (20, 93, 145), providing excellent frameworks to lead structural–functional dissections.

#### 3.4.1. The Malectin-like extracellular domain and a RALF-FER-LLG tripartite complex.

Crystal structures for insect-produced ECDs of ANXUR1, ANXUR2 (20, 93), and FER (145) reveal that the tandem MAL regions are folded into distinct domains but packed tightly against each other, separated by a linker region. These MAL domains align closely (Figure 2b,c) and, despite relatively low sequence identities, they could be superimposed readily with *Xenopus* Malectin. The dual MAL domains are arranged almost perpendicularly to each other, with the C-terminal region of MALA tightly packed against the N-terminal region of MALB. A relatively unstructured MALB C-terminal region wraps back to interact with MALA, rendering a rather rigid structure for the ECD.

The structure of an FERecd-LLG2-RALF23 heterocomplex has been solved (145). While in solution RALF23 interacted with both FERecd and LLG2 in a tripartite complex, the C-terminal side of the full-length peptide could not be detected in the complex, reflecting a flexibility consistent with an intrinsically disordered region. Therefore, the detailed structure of the heterocomplex was determined from cocrystals formed between FERecd [amino acid residues 29–423 (see Figure 2a)], pollen-specific LLG2, and an N-terminal fragment of RALF23 spanning amino acids 1–17 (145). In the heterocomplex, the N-terminal segment of RALF23 is helical and LLG2 is also mainly helical. LLG2 and RALF23 are in close proximity to MALB, although binding by the peptide and LLG2 did not impart marked conformational changes in FERecd (Figure 2b,c). The N-terminal region of RAFL23, encompassing a conserved YISY motif essential for its medium-alkalinization activity (1, 52), interdigitates into a large surface groove in LLG2. While the N-terminal fragment of RALF23 does not contact FERecd, binding studies in solution showed that the peptide was able to enhance FER binding to its in vivo partner LLG1, albeit with lower efficiency than full-length RALF23. These observations together led to the suggestion that the RALF23 N-terminal region nucleates FER-LLG1 interaction but that its C-terminal region (between amino acid residues 18–50) facilitates complex formation. With the molecular and structural perspectives established for a RALF-FER-LLG complex, it is interesting to reflect on insights already conveyed 15 years earlier that a tomato RALF associated with a 30 kDa and a 120 kDa protein (112), which are almost precisely the size of an LLG and a FER family receptor, respectively!

#### 3.4.2. Biological implications and enigmas.

Despite speculations (e.g., 6, 11, 53) and observations of the ECDs of FER and related receptor kinases interacting with the cell wall polysaccharide pectin (24, 30, 31, 76, 80), a recognizable carbohydrate-binding site could not be identified in any of the MAL domain structures (20, 93, 145). While the core of the MAL domains displayed a jelly-roll fold typical of carbohydrate-binding motifs, they all lacked the amino acid residues found in canonical carbohydrate-binding surfaces. However, several loop regions in MALA appeared to be relatively flexible, a feature noted as being used by some carbohydrate-binding motifs to bind to their sugar ligands (45, 93). Of interest is the protein–protein interface shared by the tightly packed MAL domains that is lined with highly conserved aromatic and polar residues, forming a deep cleft. The cleft has a surface area potentially accessible for interaction with a carbohydrate or a peptide, and a xylotetraose ligand could fit into the cleft formed by the juxtaposed MALA and MALB domains. The possibility of plant receptor kinases having evolved unique binding sites to sense complex plant cell wall components was left open in these structural studies (93, 145).

Many structural-functional insights have been derived from the FERecd-LLG2-RALF23 tripartite structure (145), especially when examined with point mutations related to the module and accompanied by interactions in solution examined by other biophysical methods. For instance, two mutations introduced into the MALB domain of FER independently rendered the mutant FER incapable of restoring sensitivity in *fer-4* to RALF23-inhibited growth (145). The founding point mutations in THESEUS1, *the1*-*1* and *the1*-*2*, a Gly37Asp and an Glu150Lys conversion, respectively, are in analogous locations in MALA and MALB. The mutant receptors showed a reduced capacity to suppress growth in mutants with a weakened cell wall (54). The Gly37 residue is invariant among FER family receptor kinases. In FER, a Gly41Ser change generated a temperature-sensitive allele, *fer-ts* (Figure 2a), and rendered instability to the mutant protein and root hair growth defects under restrictive temperatures (67). The Gly residue is, however, not located in an exposed surface of the MAL domain and thus is not likely to be directly involved in interacting with carbohydrate molecules. Nevertheless, that a single mutation in one of the tandem MAL domains is adequate to compromise the functions of THESEUS1 and FER attests to the functional significance of each of these domains. It also suggests that the integrity of both MAL domains together mediates a functionally important structural feature, such as their tightly packed configuration or the large cleft at their domain interface. However, RALF34 was found to bind wild-type THESEUS1 and the Gly37Asp mutant receptor comparably well, yet *the1*-*1* mutant seedlings were refractory to at least one RALF-signaled response, the RALF34-triggered rapid Ca^2+^ influx (4, 52).

Xiao et al. (145) also demonstrated that RALF interactions with potential coreceptors in vitro could be rather versatile. The RALF23 N-terminal region residues 4–17 bind LLG2, with Gly109 in LLG2 tightly juxtaposed against Ile16 of RALF23. However, biologically and based on expression profiles, RALF23-LLG1 is expected to be the functional partner, not RALF23-LLG2. Yet structural conservation between LLG1 and LLG2 prevails, allowing both to interact in vitro with a ligand that is expected to be cognate and one that is noncognate in vivo. In the immunity-suppressing mutation *llg1*-*3*, a Gly114Arg (analogous to Gly109 in LLG2) conversion resulted in the mutant GPI-AP being deficient in mediating RALF23-inhibited immunity signaling (118). In solution studies, the Gly114Arg mutation in LLG1 substantially reduced its affinity for RALF23 relative to wild-type LLG1 (145), reflecting the biological importance of the interaction between this Gly114 and the peptide ligand.

Specific and/or preferential molecular interactions between RALFs and their FER-LLG1 coreceptors also exist. For instance, RALF34 interacted with THESEUS1, whereas RALF1 did not (46), and RALF23 did not interact with the female gametophyte–specific LORELEI (145). The latter difference was attributed partially to an Arg residue in LORELEI in place of the analogous Gly123 in LLG1, which is also conserved in LLG2 and LLG3. When Gly123 in LLG1 was changed to Arg, the mutation weakened interactions with RALF23. Conversely, when the Arg residue in LORELEI was changed to Gly, it enabled binding to RALF23. However, biologically the LLG proteins appeared to be interchangeable in functional complementation studies (100). Furthermore, several RALF peptides with a conserved N-terminal segment similar to that of RALF23 bound LLG2, but a few that lack this homology did not. The distinction appears to segregate based on whether these RALFs have predictable S1P cleavage sites (129, 145) (Figure 2e).

Much remains to be uncovered in mapping key amino acid residues engaged in molecular interactions within tripartite FER-LLG-RALF complexes. Broader studies on these structures should inform where similarities and divergences lie in modules composed of varied partners mediating distinct functions.

### 3.5. The RAC/ROP GTPase Signaling Switch

In a search for molecular interactors with ROPGEF1, FER was identified as a cell surface receptor of RAC/ROPs (23, 73, 98) (Figure 2d). ROPGEFs activate these RHO GTPases (23). Activated RAC/ROPs target multiple effectors, impacting diverse response systems in the cytoplasm, including Ca^2+^, ROS, actin, microtubule, and vesicular trafficking dynamics, all fundamental components of cellular activities (29, 33, 98, 99). Many plant growth and reproductive phenotypes attributable to defects in RAC/ROP signaling are found in loss-of-FER mutants.

The FER-RAC/ROP-ROS connection has been extensively studied. For plant growth and reproduction, FER-controlled ROS production is crucial for polarized root hair growth (23), sperm release from pollen tubes in the female gametophyte (22), and a stigma gate that is responsive to and differentiates between desirable and unwanted mates (10, 24, 57, 77, 157). ANXURs and BUPSs also control the RAC/ROP-regulated ROS environment in elongating pollen tubes to ensure their integrity during growth to the target female gametophytes (7, 23). The FER-RAC/ROP-ROS pathway plays an important role in maintaining a rhizosphere microbiome that is beneficial to plants (126). There are more than 10 members in the ROPGEF and RAC/ROP protein families in *Arabidopsis* and several distinct families of effectors for the activated GTPases (29, 33, 98), including NADPH oxidases (74), which produce ROS and are ubiquitous signal mediators of myriad processes (90, 142). Thus, the RAC/ROP GTPase switch and ROS are inevitably versatile hubs for signal diversification (Figure 2d), contributing to the broad biological roles played by FER.

## 4. CELL SURFACE ELABORATORS OF THE RALF-FER-LLG CORE SIGNALING MODULE

Many molecular factors elaborate FER-LLG signaling mechanisms and diversify the actions of this core signaling module. This section focuses on molecules along the cell surface, starting with components of the extracellular matrix and at the wall–cell membrane interface and then moving to transmembrane proteins and proteins along the inner cell membrane surface.

### 4.1. Intercellular Elaborators

The cell walls secreted by neighboring plant cell protoplasts provide for an intercellular environment enriched in carbohydrates, the predominant biopolymers that make up the cell wall matrix. There is also an abundance of proteins that either are tightly associated with the cell wall or decorate the intercellular landscape as secreted protein and peptide RALFs. The extracellular domains of transmembrane proteins, such as receptor kinases, decorate the cell membrane–cell wall interface. Components from each of these milieus, which collectively are referred to as the apoplast (Figure 3), participate in regulating FER-LLG1 signaling.

#### 4.1.1. *Trans*-acting RALFs.

RALFs, as small peptides secreted to the apoplastic space, conceivably can diffuse in the extracellular milieu and act in *trans* to impact neighboring cells. The intimate interface between pollen and pistillate cells is a most fitting environment for *trans*-acting RALFs from one partner cell to impact the other, such as where the synergid cell–produced FER induces rupture of a penetrating pollen tube to enable fertilization (10, 32, 62, 64) (Figure 1a). The *trans*-acting capacity of RALFs was first suggested for RALF34 expressed in ovules because it outcompeted the *cis*-acting pollen-specific RALF4 and RALF19 for binding to the ECDs of pollen ANXURs and BUPSs (41, 42). This presumably could disengage the ANUXR and BUPS function to maintain pollen tube wall integrity, allowing the pollen tube to respond to the FER-induced environment and burst, although in vivo evidence remains lacking. Biological evidence based on multiplexed mutations of several other pollen-expressed RALFs (RALF6, RALF7, RALF16, RALF36, and RALF37) provided strong support for their acting in *trans* to mediate FER-controlled events in the pistil (162). These pollen RALFs interacted with the ECDs of pistil-expressed FER, ANJEA, and HERCULES1 and collectively enabled FER-controlled sperm release from the pollen tube in female gametophytes and polyspermy blocks during pollen tube exits from the main growth path and locally at the ovules (Figure 1b,c).

Pathogenic fungi *Fusarium* and parasitic root knot nematodes have been identified (87, 132, 144, 158). The idea that pathogen-secreted RALFs might act as signaling ligands in *trans* to bind to FER family receptors to impact host immunity response aligns with the extensively studied pattern-triggered immunity via cell surface receptors of pathogen effectors (e.g., 97, 101, 155, 156). How pathogen-produced RALFs might mediate immunity signaling warrants further investigations.

#### 4.1.2. Other *trans*-acting regulators of FER.

In *Arabidopsis*, stigma-produced RALF33 maintains a RALF33-FER-LLG1 module to mediate a tunable pollen-receptive surface in the stigmatic papillae via the core FER-RAC/ROP-ROS pathway (77) (Table 1). The pistil module maintains a basal ROS level in unpollinated stigmas. Deposition of compatible pollen, for example, during self-pollination, reduces stigmatic ROS levels within minutes. This is paralleled by pollen hydration, the initial event in pollen germination to extrude a pollen tube. Low molecular weight–secreted Pollen Coat Protein B-class peptides (PCP-Bs) (136, 137) serve as the pollen trigger, acting in *trans* to downregulate the RALF33-FER-LLG1–RAC/ROP–ROS pathway and unlocking the stigmatic gate to generate a hospitable stigmatic surface for pollen germination. These and protein–protein interaction studies led to a model in which PCP-Bs from compatible pollen outcompete stigma RALF33 for binding to FER, disengaging FER-to-ROS signaling, reducing stigmatic ROS levels to unlock the gate, and facilitating pollen hydration and germination (77).

A similar FER-controlled ROS-mediated stigmatic gating mechanism occurs in *Arabidopsis*-related but self-incompatible *Brassica rapa* (57, 157). On one hand, when pollinated by a self-compatible intraspecific relative, *B. rapa* PCP-B mediates stigmatic ROS downregulation to facilitate pollen hydration and germination. On the other hand, the FER-controlled stigmatic ROS gate is upregulated by self-incompatible pollen, blocking pollen hydration and mediating the self-incompatible response to arrest pollen germination. S-Locus Cysteine-Rich/S-Locus Protein 11 (SCR/SP11) are pollen factors that bind the stigma receptor S-Locus Receptor Kinase (SRK) (47), which controls the self-incompatibility response. SRK also interacts with FER, and cognate SCR/SP11–SRK interaction during self-incompatible pollination enhances SRK–FER interaction (Figure 3), augmenting the FER-ROPGEF-RAC/ROP-to-ROS production pathway to increase stigmatic ROS, preventing hydration and arresting germination of incompatible pollen (57, 157). Therefore, SCR/SP11 acts in *trans* via its interaction with SRK to regulate the FER-controlled stigmatic ROS gate.

Nitric oxide (NO) is an important oxygen species and is intimately related to ROS in production and signaling activity (28, 92, 141). NO also plays a critical role in regulating the FER-controlled stigmatic ROS gate (57). Compatible pollen and derived PCP-Bs trigger a rapid, transient, and FER-dependent increase in stigmatic NO, followed by NO dissipation, all in the first minutes of pollination. This parallels the time frame of pollen hydration and correlates with stigmatic ROS decline. However, incompatible pollen and their PCP-Bs cannot stimulate NO, and ROS level remains high, prohibiting pollen germination. Genetically and chemically altering the stigmatic NO condition reproduced its inverse relationship with ROS, further supporting their causal relationship. It was further established that NO suppressed FER-dependent ROS production through nitrosation of FER, which weakened its interaction with the ROPGEF-RAC/ROP switch, thus downregulating ROS production.

The interconnected FER-controlled mechanisms to differentiate desirable from unwanted mates led to efforts to genetically manipulate the stigmatic barrier in *B. rapa* (57). Downregulating FER and the FER-RAC/ROP-signaled ROS-producing enzyme NADPH oxidase in *B. rapa* pistils successfully relaxed the FER-controlled ROS-mediated stigma gate, allowing penetration by interspecific incompatible pollen into the stigma. A few of these interspecific pollen tubes managed to grow into the pistil and target the *B. rapa* ovules for fertilization, producing hybrid embryos. These proof-of-principle experiments demonstrate that mechanism-informed strategies could be successfully applied to maneuver interspecific reproduction barriers on the stigma (57).

### 4.2. Cell Wall Elaborators

Indispensable to plant life, the plant cell wall serves as the exoskeleton that maintains the integrity of each cell, the overall plant architecture, and a barrier to invasive damages such as physical wounding and pathogen penetration. The primary cell wall is malleable and responsive to dynamic cellular demands to support cell proliferation and growth and to extracellular triggers when under biotic and abiotic duress. The plant cell extracellular matrix is a complex network of carbohydrates, largely composed of cellulose, hemicellulose, and pectic polysaccharides (2, 106). It is interwoven with proteinaceous molecules that are integral structural components tightly associated with the carbohydrate matrix, such as extensins (95) and leucine-rich repeat (LRR) extensin-like (LRX) proteins (55). Enzymes that modify and degrade cell wall polymers are also in abundance to alter the physical and biochemical properties of the cell wall (19). Extracellular domains of many cell membrane–associated proteins, such as FER and LLG, and secreted molecules such as RALFs decorate the cell wall–cell membrane interface. Cell wall carbohydrates and extracellular matrix proteins that interact with and elaborate FER-LLG1 signaling are discussed (Figure 3).

#### 4.2.1. FER–pectin interaction.

Pectin is structurally and biologically the most dynamic cell wall polysaccharide, critical for cell wall structure and integrity as well as mediating its malleability to support various biological processes (2, 19, 106, 122, 143). Its polygalacturonic acid (PGA) backbone is the substrate for many pectin degradative enzymes and the source for biologically active oligogalacturonic acids, which are elicitors of diverse cellular responses (12, 19, 37, 78, 134) (Figure 3). Various molecular interaction assays showed that fragmented PGA binds the ECDs of FER and related ANXUR and BUPS (Figure 3) as well as the individual MALA and MALB domains (Figure 2a) (31, 76).

Pectin is important for cell wall strength (2, 19, 106). In the cell wall, pectin is distributed between the native methylesterified form and pectin with varying degrees of de-esterification. In the presence of divalent cations, such as Ca^2+^, in the apoplast, the free carboxyl groups from the de-esterified pectin mediate intermolecular crosslinking, impacting cell wall stiffness and penetrability (2, 19, 106, 135). Phenotypes in loss-of-FER mutants reflect a weakened cell wall. For example, root hairs in *fer* seedlings leak cytoplasm and collapse (23, 74) (Figure 1g) and roots have reduced mechanical strength, making them less able to penetrate growth barriers than their wild-type counterparts (120). When seedlings encountered high salinity, the *fer* root cells exploded, terminating root growth, while wild-type cells swelled but remained intact and growth recovered upon acclimation to salt-induced stress (31) (Figure 1g).

In the ovules, loss of FER results in the loss of de-esterified pectin at the entrance to the female gametophyte, accompanied by the relaxation of the polyspermy block resulting in the multiple-pollen-tube-penetration phenotype observed in *fer* mutants (10, 24). Single pollen tube entry is dependent on not only FER but also pectin, which, together with an arriving pollen tube, triggers NO accumulation at the local polyspermy block in the ovule (Figure 1d). NO modifies the pollen tube attractant LURE (130), inhibiting its activity and preventing its further secretion from the target synergid cells. This engages the ovular polyspermy block to prevent late-arriving pollen tubes from entering an already penetrated ovule (24). Therefore, the FER-controlled process is likely the result of pectin-regulated cell wall penetrability in response to abrupt [Ca^2+^] changes upon the arrival of the first pollen tube and pectin-derived fragments as biologically active agents, triggering NO production and the NO-induced biochemical and cellular consequences (12, 19, 37, 78, 134).

FER plays a crucial role in mechanosensing (83, 84). De-esterified pectin binding to FERecd stimulates the activation of RAC/ROP (76, 131). This interaction apparently impacts a major cellular target system of RAC/ROPs, the microtubule cytoskeleton, and underlies the mechanical stress sensing encountered during pavement cell morphogenesis. However, results in other studies uncoupled the FER-regulated microtubule response to stress from mechanosensing by FER, leading to the suggestion that the two FER-regulated phenomena might function in parallel. For the RALF4/RALF19-ANXUR/BUPS-LLG2/LLG3-controlled pollen tube integrity, LLG2/LLG3-deficient pollen tubes showed aberrant pectin deposition patterns, consistent with a weakened cell wall prone to rupture (30). BUPS1-mediated mechanical sensing might also contribute to pollen tube integrity during growth that transitions from the stigmatic tissue to the transmitting tissue (163).

#### 4.2.2. FER interaction with cell wall–associated LRX.

Extensins are hydroxyproline-rich glycoproteins important for wall structure and integrity (95). They tightly associate with the cell wall matrix via extensive inter- and intramolecular crosslinking. LRXs are chimeric extensins with a variable stretch of LRRs N-terminal to their extensin domain (55, 109). Like extensins, LRXs are extremely resistant to extraction from the cell wall. Participation in FER and related signaling modules by LRXs was first implicated via their association with pollen-expressed RALF4 and RALF19 (27, 88). A triple mutant in pollen-expressed LRX8, LRX9, and LRX11 showed reduced pollen tube growth rates. Protein interaction, functional assays, and cocrystallization of LRX8-RALF4 (94) established a LRX-RALF partnership.

Physical and functional LRX–RALF interactions have also been established for several vegetative cell–expressed LRXs and RALFs (56, 160, 161). *lrx3 lrx4 lrx5* triple-mutant seedlings resemble *fer* morphologically: Both are growth-inhibited and display hypersensitivity to high salt. However, unlike *fer* mutants, which are sensitive to RALF1-inhibited growth, the triple-*lrx* mutant seedlings remained resistant (25). The LRR domains, that is, without the extensin region, of the LRXs physically interact with FERecd in protein pull-down assays and yeast two-hybrids (25), and they co-immunoprecipitated when expressed in plant cells (56). Biological studies suggest functional connections between LRX and FER, mediating sensing of mechanical stresses at the cell wall from increased turgor pressure from vacuolar expansion during root cell growth (25) and seedling growth (56). LRXs lack a cytoplasmic domain for signal propagation. Functional connection with FER and interaction with RALFs both provide a linkage whereby extracellular sensing by LRXs could be passed on for signal propagation to the cytoplasm via transmembrane receptor kinases. However, the LRX-interacting RALF1 did not affect co-immunoprecipitation of FERecd and the LRX-LRR domains (56). How a tripartite interaction might be mediated remains puzzling. The pollen RALF-LRX structural study reported that tertiary complexes with LRX, RALF, and LLG2 or LRX, RALF, and a FER family receptor kinase have not been observed in crystallographic and related biochemical studies (94). It suggested that in the pollen tube the RALF–LRX and RALF–LLG2/LLG3 interactions might function in two parallel but distinct pathways. One is with RALF-LRX controlling processes outside the tube cell and the other is RALF-LLG2/LLG3 via interaction with ANXURs for signaling inside the tube through an effector protein called MARIS, which is part of the cellular machinery for redox regulation (5) (see Figure 3). It will be very interesting to learn about the precise molecular bases that underlie the functional connection between LRXs and the FER family receptor kinases.

#### 4.2.3. FER sensing of perturbations originating from the secondary cell wall.

Lignified secondary cell walls provide more rigidity in nonexpanding cells and cells requiring added mechanical wall strength, such as those in the trachea (2). Recent studies suggested a role for FER in sensing perturbations that originated from compromised secondary cell walls. Secondary wall biosynthetic mutants in *Arabidopsis* produced a higher level of soluble pectin-derived elicitors active in inducing pathogen-related (PR) gene expression (36). To uncover a molecular linkage between these pectin elicitors and surface receptors, and using PR gene expression as a readout, Liu et al. (78) showed that FER is important for the capacity of the lignin biosynthetic mutant *ccr1*-*3* to produce these biologically active elicitors. Introducing *fer-4* into *ccr1*-*3* resulted in qualitative changes in soluble pectin fragments, which was attributed to *fer-4* suppressing the expression of a polygalacturonase, a pectin-degrading enzyme (36). A functional model connecting the secondary cell wall to the sensing of pectin aberrations (78) shows FER playing a critical role in bridging the impact of lignin status on the production of soluble pectin elicitors from the primary cell wall to trigger a downstream defense response. The model suggests that FER senses alterations in the cell wall and regulates the production of cell wall–remodeling enzymes, impacting pectin composition, extractability, and the release of biologically active oligosaccharide elicitors. These elicitors in turn might be perceived by other receptors, such as the wall-associated kinases (WAKs), though the signal-sensing mechanism for these pectin-binding receptor kinases remains unclear (70).

### 4.3. Cell Membrane–Associated Elaborators

Transmembrane proteins and cytoplasmic proteins recruited to the cell membrane level are frequent components of cell surface signaling modules. Here we discuss examples of such molecules that integrate into the FER-LLG1/LORELEI signaling schemes.

#### 4.3.1. Powdery Mildew Resistance Locus O 7/NORTIA: a booster of FER signaling with a regulatory role in synergid cell Ca^2+^ dynamics.

Powdery mildew resistance locus Os (MLOs) are important regulators for disease susceptibility to the fungal pathogen powdery mildew and have been broadly studied in a large variety of crop species (61, 71). MLOs are seven transmembrane-domain proteins with an extracellular N-terminal domain, three cytoplasmic domains, and a C-terminal cytoplasmic stretch (Figure 3). MLOs were first connected to FER signaling when *Arabidopsis* defective in an ovule-expressed MLO7/NORTIA was found to display a pollen tube nonbursting phenotype similar to that in *fer* and *lorelei* (66). The MLO7 discovery also provided the first implication of FER in the plant disease defense landscape. The pollen tube bursting defects, however, only occurred in ~20% of the *mlo7* mutants, considerably lower than the ~80% typically observed in *fer* and *lorelei* mutants. The synergid cell–expressed MLO7-GFP was located intracellularly in various secretory organelles before pollen tube arrival at the female gametophyte. With the arrival of a pollen tube, MLO7-GFP became polarized and was translocated to the thickened synergid cell wall region, which also has a high density of innervating synergid cell membranes. The thickened cell wall region, referred to as the filiform apparatus (Figure 1a), marks the entrance of the female gametophyte where FER and LORELEI also concentrate. This translocation process failed in *fer* mutant ovules. A calmodulin-binding domain occurs in the C-terminal region of MLOs, suggesting participation in Ca^2+^ signaling–related events (61, 66, 71). A signature cytoplasmic Ca^2+^ oscillatory pattern in the target synergid cell of an approaching pollen tube was disrupted in *fer*, *lorelei*, and *mlo7* mutant female gametophytes (60, 96). Taken together, these observations led to the suggestion that MLO7 acts as a booster (63) to FER-LORELEI-signaled sperm release from the invading pollen tube (Figure 3).

Mechanistic connections to MLO regulation of Ca^2+^ dynamics have been pursued ever since their discovery (61, 66, 71). A recent study concluded that MLO7 functions as a Ca^2+^ channel complexed with FER-LORELEI (40) to mediate pollen RALF4- and RALF19-triggered Ca^2+^ influx in the synergid cells. While many questions remain, results implicating MLOs functioning as Ca^2+^ channels were further extended to the pollen tube–located RALF-ANXUR/BUPS-LLG2/LLG3 modules (39). Given the importance of MLOs in defense (61, 71) and contributions from the FER family receptor kinases (101, 159) in immunity signaling, whether an MLO-Malectin domain kinase connection extends beyond signaling modules in reproductive events will be important to examine, and if so, whether it also aligns with MLOs functioning as a Ca^2+^ channel.

#### 4.3.2. Transmembrane receptor kinase and receptor-like cytoplasmic kinase elaborators.

Using several pathogenesis systems, including the model *Pseudomonas syringae*–*Arabidopsis* system, and the well-established elicitors of immunity responses elf18 and flg22, Stegmann et al. (129) provided molecular and functional evidence for FER-LLG1 in immunity signaling in response to bacterial and fungal pathogens. The LRR receptor kinases Flagellin-Sensing 2 (FLS2) and Brassinosteroid Insensitive 1 (BRI1)-Associated Kinase 1 (BAK1) function as a coreceptor pair for immunity signaling (48, 101). Screening for suppressors of the immune response–compromised *bak1*-*5*, Stegmann et al. (129) identified a missense mutation in S1P, which is known to be critical for processing prepro-RALFs into mature secreted RALFs (128) (Figure 2e). *s1p* mutants displayed elevated immunity responses relative to wild type, ranging from the rapid immunity readout response of a ROS burst within minutes of elicitor application to higher resistance to pathogens upon infection. RALF23, whose prepro-peptide is a substrate for S1P (128) (Figure 2e), was determined to function as a signaling ligand binding to the FERecd to suppress immunity responses. Additional RALFs might also participate in a similar capacity.

Mechanistically, Stegmann et al. (129) determined that FER interacts weakly with BAK1 and FLS2 (Figure 3), and elicitors stimulated FER–BAK1 but not FER–FLS2 interaction. The elicitor-enhanced coreceptor FLS2–BAK1 interaction was suppressed in *fer* mutants, revealing FER as a facilitator of immunity response. Elicitor stimulation of the FLS2–BAK1 interaction was inhibited by RALF23 application or RALF23 overexpression. These results led to the suggestion that FER functions as a scaffold, perhaps in membrane microdomains, to regulate the assembly of an immune receptor complex (48, 129). RALF1 also binds BAK1 and increases its phosphorylation status (17). BAK1 is also a coreceptor of BRI1, the receptor for BR, and RALF1 is known to antagonize several BR-regulated growth responses (3, 17). These together would suggest that BRI1 could also be recruited to the FER scaffold under certain cellular or apoplastic conditions.

Receptor-like cytoplasmic kinases (RLCKs) are closely related to transmembrane receptor kinases but lack a transmembrane domain and an ECD for extracellular signal sensing (123). Several RLCKs that interact prevalently with FER family receptors (e.g., 18), including those with important roles in immunity signaling, have been recently reviewed in detail (101, 159). MARIS, the redox regulatory RLCK, interacts with ANXURs and FER in pollen tubes and root hairs (5) (Figure 3). That RLCKs interact directly with the cytoplasmic domain of FER family receptors further fosters the model of FER functioning as a scaffold, assembling cytoplasmic signaling molecules to expand downstream signaling diversification (101, 129, 159).

## 5. CYTOPLASMIC AND NUCLEAR ELABORATORS

Many studies demonstrated FER involvement in intertwining cytoplasmic and nuclear pathways (75, 101, 159). Highlighted here are recent studies that delineate how the well-established pathways of light signaling via phytochrome B (PhyB) and stress hormone signaling via the transcription factor MYC2 contribute to the broader biological roles of FER. Emerging data linking FER to TARGET OF RAPAMYCIN (TOR) and autophagy, major cell growth, and cellular homeostasis regulatory systems are also discussed in this section. It is worth noting here that while not all FER-regulated processes rely on the kinase activity in FER (see 21), FER kinase activity is important for the phosphorylation of PhyB and MYC2, which are important nodes for signal diversification.

### 5.1. Elaborating Through Phytochrome, Connecting FER-Controlled Growth, and Stress Signaling

To further the mechanistic understanding of how LRXs mediate salt-stress management (161), Liu et al. (82) identified *phyB-9*, an often-used mutant allele of the red/far-red photoreceptor PhyB (117), as a suppressor of salt stress hypersensitivity in *lrx3 lrx4 lrx5* triple mutant seedlings. The *phyB-9* mutation also suppressed many of the phenotypes in *fer-4* under normal or salt-stressed growth conditions. These results uncovered a functional connection between light and FER signaling and their interconnected impacts in regulating growth and mediating stress responses.

Mechanistically, FER was found to be important for the dynamic shift between the inactive (Pr) and active (Pfr) forms of PhyB. Light induces cytoplasmic PhyB to translocate to the nucleus, forming PhyB protein bodies (referred to as photobodies) (51) (Figure 3), accompanied by the photoconversion of Pr to Pfr. Pfr interacts with downstream transcription factors to activate photomorphogenesis. Return to the dark induces disassembly of the photobodies, accompanied by the conversion of Pfr back to Pr and the reduction of the nuclear PhyB level. In vitro, the FER kinase domain interacts directly with and phosphorylates PhyB and accelerates the conversion of Pfr to Pr. Salt stress inhibited FER kinase activity and retarded the light-regulated, photobody-associated processes. Interestingly, pectin status, in particular de-esterified pectin, is also an important factor in FER-regulated salt stress response (44). Given this evidence, Liu et al. (82) suggested that a FER–cell wall–sensing linkage mediates the salt stress signal to the phytochrome system. While many details will likely emerge, these results together paint a picture of FER playing a role in coordinating light-signaled, phytochrome-mediated growth promotion with salt stress–induced growth inhibition, underscoring the importance of FER in keeping a balance between growth and survival when under stress.

### 5.2. Elaborations Through Transcription Factors

Results from a series of transcriptomics, proteomics and phosphor-proteomics studies suggest that direct interaction with diverse transcription factors is a major component underlying the intersection of FER with stress signaling (50, 139). Distinct sets of transcription factors were identified in these studies, affecting various stress response pathways, such as those signaled by the stress hormones jasmonic acid (JA) and ABA. MYC2, a master transcription factor of JA signaling, controls a cascade of other transcription factors culminating in suppression of defense-related genes (50). Functional studies demonstrated that loss of FER increased the accumulation of JA-regulated transcripts in *fer* mutant plants and their susceptibility to the bacteria pathogen *P. syringae*, establishing that FER positively regulates immunity by downregulating JA signaling. Mechanistically, the FER kinase domain interacts directly with MYC2 in vitro and in vivo, phosphorylating and destabilizing MYC2 and reducing its nuclear accumulation. This in turn alleviates MYC2 suppression of the immunity response (139) (Figure 3).

Phosphoproteomic analysis showed that several ABA-induced transcription factors, including ABI5 (Figure 3), were hypophosphorylated in *fer* mutant plants (139). ABA suppresses the growth of germinating seedlings, and ABI5 inhibits cotyledon greening. Germinating *fer* mutant seedlings are hypersensitive to ABA, which causes the cotyledons to turn yellow more readily than wild-type seedlings (74, 152) (Figure 1i). Guo and colleagues (139) showed that FER phosphorylates ABI5 and renders the transcription factor more prone to degradation, thus keeping its capacity to inhibit cotyledon greening in check. Accumulation of hypophosphorylated ABI5 thus accelerated cotyledon yellowing in *fer* seedlings. This and the FER activation of ABI2, the protein phosphatase 2C that negatively regulates ABA signaling via RAC/ROP–ABI2 interaction (152), are likely to work in concert to mediate ABA-regulated responses.

### 5.3. Connecting FER to TOR Signaling and Autophagy

TOR is a conserved protein kinase that integrates signaling and the metabolic network, and controls almost all aspects of life, from growth and development to stress management (119). Recent evidence supports that RAC/ROPs are linked to TOR signaling in *Arabidopsis* (113). Loss of FER induces strong metabolic defects, for example, rendering an overaccumulation of starch throughout developing seedlings (69, 148). Given that FER is an upstream regulator of RAC/ROPs, the pleiotropic phenotypes encompassing, for example, growth, development, stress-related, and metabolic defects in *fer* mutants are consistent with recent studies that link FER to TOR (102, 125, 140). Under normal plant growth conditions, loss of FER rendered moderately increased sensitivity to the inhibitor of TOR kinase AZD8055 relative to wild type (102, 140). Using phosphorylation of S6K protein, a routinely used readout for TOR kinase activity (119), these studies showed that loss of FER resulted in reduced TOR activity.

TOR is known to function upstream of autophagy (14), a cellular degradative process to remove nonfunctional cellular components under various conditions of need. Autophagosomes are organelles that process cellular contents destined for degradation during autophagy. Based on autophagosome numbers, *fer-4* seedlings harbor significantly elevated autophagic activity relative to wild type under normal growth and when starved for sugar, which augments autophagy, consistent with the idea that FER negatively regulates autophagy (140).

## 6. CONCLUDING REMARKS

The discussion here could not do justice to all the studies that have been reported since the discovery of FER as a receptor kinase in 2007 (26), nor the underlying signaling complexities of FER-controlled processes. Together these studies firmly establish that FER and the FER family receptor kinases are critical regulators that enable plants to grow, proliferate, and cope with their ever-changing environment. My hope is that this abbreviated summary of the components that are core to FER signaling and those that elaborate and regulate its signaling capacity suffices to provide an overview of how, under different functional contexts, FER and its related receptor kinases could be shaped on many levels to meet biological demands. Complexity is a biological reality; the already overwhelming landscape of the FER signaling network (e.g., 15, 32, 75, 101, 129, 159) exemplifies this. In the future, research will need to look beyond those immediate next questions prompted by published findings to address broader challenges that refine our mechanistic understanding of the FER family receptors and explore emerging opportunities, in particular, translational efforts in agriculturally and environmentally important plant species.

## Figures and Tables

**Table 1:** Core components of RALF-FER-LLG signaling modules from *Arabidopsis*

	Receptor	Coreceptor	Ligand	Main function	Reference(s)
Variations of the RALF-FER-LLG module^a^					
Vegetative tissues	FER	LLG1	RALF1, RALF23	Growth and immunity	23, 74, 129, 145
	THESEUS1	Not reported	RALF34	Cell wall sensing; growth and lateral root emergence	46, 54
	LETUM2	LLG1	Not reported	Cell death in immunity response	58
Pistil: stigma	FER/ANJEA	LLG1	RALF33/RALF23^b^	Stigma gating to control pollen germination	77
Pistil: ovule/female gametophyte	FER ANJEA/HERCULES1^c^	LORELEI	Not reported	Pollen tube bursting to release sperm for fertilization; prevents polyspermy	22, 35
Pollen	ANXUR1/ANXUR2^c,d^ BUPS1/BUPS2^e^	LLG2/LLG3^c^	RALF4/RALF19^c^	Pollen tube integrity: prevents bursting during growth in the pistil	30, 41, 43
Intercellular interactions of the RALF-FER-LLG module					
Cell-cell interface: pollen-stigma	FER in stigma	LLG1 in stigma	Pollen Coat Protein B-class peptides (PCP-Bs)	Pollen triggers to unlock the stigma gate and facilitate pollen germination	57, 77
Cell-cell interface: pollen-ovule	FER in ovule	LORELEI	Pollen RALF6, RALF7, RALF16, RALF36, RALF37	Mediates pollen tube bursting and prevents polyspermy	162
	FER in ovule	LORELEI	Pollen RALF 4, RALF19	Triggers Ca^2+^ influx in synergid cells	40

a The FERONIA (FER) protein family has 17 members, the LORELEI-like glycosylphosphatidylinositol-anchored protein (LLG) protein family has 4 members, and the RAPID ALKALINIZATION FACTOR (RALF) protein family has >35 members (1, 32, 100).

b RALF33 and RALF23 act similarly.

c Functioning redundantly.

d Function as obligatory ANXUR/BUPS heteromers.

e BUPS1 is almost exclusively functional; loss of BUPS2 alone has no phenotype.

## SUMMARY POINTS

1. FERONIA (FER) receptor kinase partners with glycosylphosphatidylinositol-anchored protein (GPI-AP) LLG1 or LORELEI to form a coreceptor pair, and its activity is regulated by the peptide-signaling ligands termed RAPID ALKALINIZATION FACTORS (RALFs). Initially identified as crucial for female fertility in *Arabidopsis*, FER has emerged as a global regulator that profoundly affects plant growth and survival.
2. FER, LLG, and RALFs are from multiple protein families conserved across plant species. Modules assembled from differentially expressed members of these three protein families harness tremendous signaling potential. Only a few from *Arabidopsis* have been functionally and mechanistically examined.
3. FER plays a critical role in mediating female contributions to the reproductive process; FER-related receptors are critical counterparts in pollen mediating male contributions to pollen-pistil interactive events during reproduction.
4. FER intersects with pathways controlled by major growth and stress hormones, including those of auxin, brassinosteroid, ethylene, abscisic acid, and jasmonic acid.
5. Loss-of-function *fer* mutants are highly pleiotropic, and resulting phenotypes ranging from whole-plant and specific cell growth defects to compromised defense responses to biotic and abiotic stresses are readily notable.
6. Mechanistic studies uncovered RAC/ROP GTPases and reactive oxygen species as major signal mediators and identified diverse molecules from the extracellular matrix, cell membrane, cytoplasm, and nucleus that interact with the FER-LLG1 module and elaborate the FER signaling network.
7. Transcriptomics and proteomics studies uncovered multiple layers of potential participants in the FER signaling network.
8. High-resolution structures of the extracellular domains of FER and ANXURs and of a tripartite RALF-FER-LLG complex are available, providing a baseline at the atomic level for a precise structural-functional understanding of key interactions between the core components of various RALF-FER-LLG modules and their cast of collaborators.

## FUTURE ISSUES

1. Foundational knowledge of FER and related receptor kinases is still limited. The rapid and expansive rise in FER-related studies has not had the benefit of intensive efforts to build depth or provide validation and refinement of existing data and hypotheses. Careful analysis of the mounting information that has already emerged to fill the FER functional and mechanistic landscape will be critical for a productive next phase.
2. How FER achieves its biological promiscuity is still a major puzzle, though insights are emerging (80). Conceivably, each of the core components contributes to the complexity of FER biology. RALF peptides have intrinsically disordered regions, which facilitate structural versatility for molecular interactions. The capacities of FER to act as a scaffold for molecular assemblies on the cell surface and as a protein kinase for signal propagation in the cytoplasm can generate functional diversification. As GPI-APs are mostly located in membrane microdomains, the LLGs could be the lynchpins to mediate interactions between FER-LLG and other signaling systems. While promising to be multifaceted, major unifying themes among these facets may be identifiable, and, if so, the knowledge may illuminate paths to resolve how these signaling schemes might be interconnected.
3. Molecular mechanisms explaining the underlying functional and physical interactions between FER family receptor kinases and cell wall polysaccharides and other proteinaceous components are needed to dissect these interactions and understand how each contributes to their diverse sensing roles, which range from detecting molecular signals to mechanical stress.
4. Evidence of FER interaction with lipid components of the cell membrane is emerging (124) and requires further investigation.
5. Evidence for physical and functional interactions between FER and other regulators in the cell membrane is strong, but how these interactions are organized and how their dynamics on the cell surface are regulated are not clear. Recruitment into membrane microdomains is in principle undisputable, but experimental evidence will likely be best achieved from multiple research approaches.
6. Many well-established protein mediators are already linked, not only functionally but also physically, to FER and related receptors. The depth of functional knowledge in plant genes is a major asset in identifying the key functional hubs and differentiating them from noise. It will take stepwise and communal efforts to identify these hubs and eventually generate a precise map of molecules that functionally interact with the FER family receptor kinases.
7. FER-related studies have already extended into major crop species (57, 157). Mechanistic concepts advanced from studies in model systems will be rich grounds on which to build for translational efforts to improve how crop species might cope with environmental challenges, and, perhaps equally important, to seek portals for plants to improve the environment.

## Footnotes

1 CrRLK1 is the first of the family members whose sequence was determined (116); there have not been any follow-up studies.

2 The founding mutations *fer-1*/+ and *sirène*/+ were originally considered female sterile. Many FER alleles have been reported since, and homozygous *fer/fer* plants were identified later; among these, *fer-4* was used in most studies. *fer* is used for general discussion.

3 Self-fertilized *fer/fer* plants produce about 20% of the seeds produced by wild-type plants. Approximately 80% of ovules from *fer/fer* (40% from *fer-1*/+) plants are sterile because mutant female gametophytes fail to burst pollen tubes to release sperm for fertilization.

4 Transcriptomics databases show that FER expression typically is more prominent than that of coexpressing members of the family; in pollen, five FER family members are expressed, four of which at very high levels.

## Abbreviations

THESEUS1: a FER family receptor kinase named for a hero from Greek mythology who slayed villains
LORELEI: member of the LLG family named for a siren who lured sailors to their doom at the edge of the river Rhine
ANXUR: a FER family receptor kinase named for the male consort of the goddess Feronia
Buddha’s Paper Seal (BUPS): a FER family receptor kinase named from a plot in the famous Chinese mythical tale *Journey to the West*
HERCULES: a FER family receptor kinase named for a famous hero of Greek and Roman mythology
ANJEA: a FER family receptor kinase named for a fertility goddess in Aboriginal mythology
ERULUS: a FER family receptor kinase named for the son of Feronia
LETUM1: a FER family receptor kinase named for the Roman personification of death
NORTIA: named for an Etruscan goddess of fertility
